# Best Procedures for Leaf and Stem Water Potential Measurements in Grapevine: Cultivar and Water Status Matter

**DOI:** 10.3390/plants12132412

**Published:** 2023-06-22

**Authors:** Martina Tomasella, Alberto Calderan, Alenka Mihelčič, Francesco Petruzzellis, Riccardo Braidotti, Sara Natale, Klemen Lisjak, Paolo Sivilotti, Andrea Nardini

**Affiliations:** 1 Department of Life Sciences, University of Trieste, via L. Giorgieri 10, 34127 Trieste, Italy; martina.tomasella@units.it (M.T.); alberto.calderan@uniud.it (A.C.); fpetruzzellis@units.it (F.P.); sara.natale@phd.units.it (S.N.); 2 Department of Agricultural, Food, Environmental and Animal Sciences, University of Udine, Via delle Scienze 206, 33100 Udine, Italy; braidotti.riccardo@spes.uniud.it (R.B.); paolo.sivilotti@uniud.it (P.S.); 3 Department of Fruit Growing, Agricultural Institute of Slovenia, Viticulture and Enology, Hacquetova Ulica 17, SI-1000 Ljubljana, Slovenia; alenka.Mihelcic@kis.si (A.M.); klemen.Lisjak@kis.si (K.L.); 4 Department of Biology, University of Padova, Via Ugo Bassi 58B, 35121 Padova, Italy

**Keywords:** *Vitis vinifera*, leaf water potential, leaf sampling, water status, stem water potential, pressure chamber

## Abstract

The pressure chamber is the most used tool for plant water status monitoring. However, species/cultivar and seasonal effects on protocols for reliable water potential determination have not been properly tested. In four grapevine cultivars and two times of the season (early season, Es; late season, Ls, under moderate drought), we assessed the maximum sample storage time before leaf water potential (Ψ_leaf_) measurements and the minimum equilibration time for stem water potential (Ψ_stem_) determination, taking 24 h leaf cover as control. In ‘Pinot gris’, Ψ_leaf_ already decreased after 1 h leaf storage in both campaigns, dropping by 0.4/0.5 MPa after 3 h, while in ‘Refosk’, it decreased by 0.1 MPa after 1 and 2 h in Es and Ls, respectively. In ‘Merlot’ and ‘Merlot Kanthus’, even 3 h storage did not affect Ψ_leaf_. In Es, the minimum Ψ_stem_ equilibration was 1 h for ‘Refošk’ and 10 min for ‘Pinot gris’ and ‘Merlot’. In Ls, ‘Merlot Kanthus’ required more than 2 h equilibration, while 1 h to 10 min was sufficient for the other cultivars. The observed cultivar and seasonal differences indicate that the proposed tests should be routinely performed prior to experiments to define ad hoc procedures for water status determination.

## 1. Introduction

The assessment of plant water status via water potential measurements is of pivotal importance in many research studies focused on basic and applied plant sciences. Despite the availability of several methods based on direct or indirect approaches (e.g., [1,2,3,4]), the pressure chamber is still the most widely available and employed instrument for the accurate and cost-effective monitoring of plant water status. This is because it is relatively cheap and easy to use, as its principle is based on a simple measurement of the pressure of gas (nitrogen, air) inflated in a sealed chamber [5].

The pressure chamber represents a useful tool for assisting irrigation scheduling in crops, in order to optimize yield and quality of the product [6]. For example, the minimum leaf water potential (Ψ_leaf_) and stem water potential (Ψ_stem_) of grapevine strongly correlate with berry size and yield [7]. In this context, a study conducted on 10 vineyards cultivated in the Classical Karst with the same cultivar showed that grapevine water status can be highly heterogeneous, even within small spatial scales [8], due to minor geomorphological differences coupled with variations in microclimate and rooting depth [9,10]. The large spatial variability of plant water status is not uncommon, even within single vineyards, calling for the need for measurements on a large number of samples to obtain a reliable picture of the actual stress levels and water needs. Water potential monitoring of large numbers of leaves is time-consuming, and realistically not possible to be completed within narrow time intervals [11]. However, the most informative values of plant water potential are measured in narrow time frames, such as at predawn (Ψ_pd_), or within about 1–2 h around midday for minimum Ψ_leaf_ and Ψ_stem_. Hence, some researchers optimize sampling by collecting a large number of leaves in a narrow time interval and storing them until water potential measurements, trying to minimize water loss from sample collection to actual measurement [12]. The reliability of this approach, and/or the magnitude of eventual artefacts in water potential estimates, are still poorly known.

In a recent study, several assumptions for correct pressure chamber measurements have been tested for some species [13]. First of all, avoiding water losses upon leaf harvest for water potential measurements is mandatory for precise and accurate water potential determination, as leaf water loss can cause a significant drop in water potential in a very short time [11], and also depends on vapour pressure deficit (VPD, [12]). In this respect, tests on appropriate storage conditions indicate that leaves should be tightly wrapped in cling film and preserved with moist paper in zip-lock plastic bags, to be maintained in the dark in refrigerated bags until measurement [13,14].

Regarding Ψ_leaf_ sampling procedures, it has been pointed out that a certain equilibration time after leaf excision could be needed before the Ψ_leaf_ determination of transpiring leaves [13]. This is because transpiring leaves display a water potential gradient between leaf xylem and more distal regions such as epidermal cells, and equilibration is required to dissipate such gradients and allow for correct estimates of average water potential [15]. On the other hand, storing leaves for long time intervals might lead to uncontrolled water loss and the incorrect determination of plant water status. Exact equilibration times and maximum possible storage times might be species-specific or cultivar-specific, and might also change on a seasonal basis due to morphoanatomical modifications of the leaves [16]. Moreover, the water status (i.e., the actual water potential) of the plant might determine the Ψ_leaf_ variation over leaf storage time due to the different leaf capacitance before and after the turgor loss point [17]. All these factors could affect the Ψ_leaf_ measurement reliability, accounting for a decreased accuracy, as suggested by a meta-analysis on grapevine [18].

Stem water potential (Ψ_stem_) is a parameter commonly used for water management and irrigation scheduling in several fruit trees [19,20], with predetermined target levels, below which irrigation is desirable. In grapevine, these thresholds can be adjusted to modulate berry and wine quality (e.g., [8,21,22]). Regarding Ψ_stem_ measurements, bagging leaves prior to collection is required to allow for equilibration of Ψ_leaf_ with Ψ_stem_. In potted seedlings dehydrated in the laboratory under low irradiance, Ψ_stem_ equilibration is almost instantaneous for some species, except at very low water potentials when xylem embolism and possible hydraulic disconnection between leaf cells can decouple Ψ_leaf_ and Ψ_stem_ [13]. However, only a few tests of the necessary equilibration times have been performed under field conditions, i.e., in a *Quercus rubra* tree [23], in three orchard fruit trees [19,24] and in late-season conditions in two grapevine cultivars [11]. Variations in soil water availability and in atmospheric VPD modify stomatal aperture, with impacts on transpiration that might influence the difference between Ψ_leaf_ and Ψ_stem_ [19], thus leading to different equilibration times needed for accurate Ψ_stem_ determination. Moreover, stomatal sensitivity to water stress has been shown to widely vary, depending especially on scion–rootstock combination [25]. Yet, to our knowledge, no experiment has tested possible cultivar or seasonal/drought stress effects on the minimum equilibration time required for accurate Ψ_stem_ determination in the field.

In this study, we tested two protocols for accurate Ψ_leaf_ and Ψ_stem_ determination in four different grapevine cultivars, in two different times of the growing season (late spring and late summer), corresponding to different phenological phases and stress levels (low and moderate-to-severe, respectively). We specifically aimed at characterizing (i) the maximum storage time allowing reliable Ψ_leaf_ measurements and (ii) the minimum equilibration time required to correctly estimate Ψ_stem_.

## 2. Results

### 2.1. First Test: Time Span from Leaf Sampling to Water Potential Measurements

**Figure 1:**
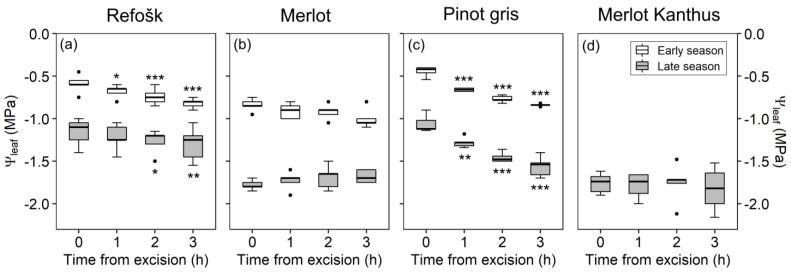
Testing time from leaf sampling to water potential measurements in (**a**) Refošk; (**b**) Merlot; (**c**) Pinot gris; (**d**) Merlot Kanthus^®^ grapevine cultivars. Median values, 25th and 75th percentiles of leaf minimum water potentials (Ψ_leaf_) measured at different timings from leaf excision in the early season (end May 2020) and in the late season (end August–beginning of September 2020/2021). Asterisks indicate significant differences between Ψ_leaf_ at 0 h (control) and the other storage times (* 0.01 < *p* < 0.05, ** 0.001 < *p* < 0.01, *** 0.001 > *p*; *n* = 5). Dots indicate outliers.

In the end of May 2020 (early-season campaigns), cv. ‘Refošk’ showed an average Ψ_leaf_ of −0.59 MPa, as measured immediately after sampling. Significant progressive drops in Ψ_leaf_ were observed in leaves stored for 1 h (by 0.09 MPa on average), 2 h (by 0.15 MPa) and 3 h (by 0.23 MPa) before measurements (Figure 1a, Table S1). In ‘Pinot gris’, a stronger and significant drop of 0.22 MPa was already recorded after 1 h of storage, compared to an immediate Ψ_leaf_ determination (from −0.44 MPa to −0.66 MPa), and it further decreased to about −0.84 MPa after 3 h (Figure 1b). Conversely, in ‘Merlot’, there was no significant variation in the measured Ψ_leaf_ upon leaf storage, although after 3 h of leaf storage, it tended to be slightly (by 0.16 MPa) lower than at 0 h (*p* = 0.052, Table S1).

At the end of the season, Ψ_leaf_ in all vineyards was lower than that measured in early-season campaigns. For the cvs. ‘Merlot’ and ‘Merlot Kanthus^®^’, storage time did not affect the measured Ψ_leaf_, with average values of −1.78 MPa and −1.76 MPa, respectively (Figure 1b,d). On the contrary, as observed in spring, average Ψ_leaf_ of ‘Pinot gris’ significantly decreased as a function of storage time, from −1.06 MPa at time 0 to −1.28 MPa, −1.46 MPa and −1.56 MPa at 1, 2 and 3 h after leaf collection, respectively (*p* < 0.001 for all comparisons, Table S1). Ψ_leaf_ of ‘Refosk’ did decrease 2 h and 3 h after leaf collection, by about 0.11 MPa (*p* = 0.027) and 0.14 MPa (*p* = 0.006) with respect to time 0, respectively.

### 2.2. Second Test: Equilibration Time for Stem Minimum Water Potential Measurements

**Figure 2:**
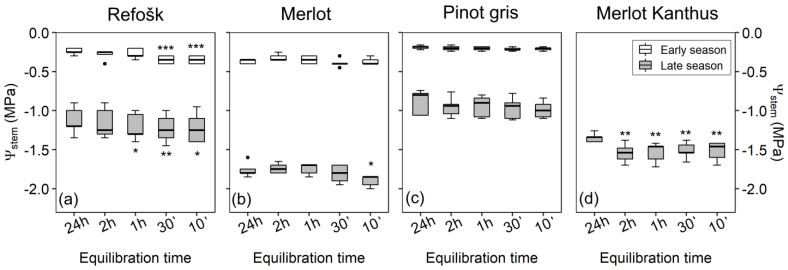
Testing equilibration time for stem minimum water potential (Ψ_stem_) measurements in (**a**) ‘Refošk’; (**b**) ‘Merlot’; (**c**) ‘Pinot gris’; (**d**) ‘Merlot Kanthus^®^’ grapevine cultivars. Median values, 25th and 75th percentiles of Ψ_stem_ measured in grapevine leaves with different equilibration times in the early season (end May 2020) and in the late season (end August–beginning of September 2020/2021). Asterisks indicate significant differences between Ψ_stem_ measured for 24 h equilibrated leaves (control) and the other storage times (* 0.01 < *p* < 0.05, ** 0.001 < *p* < 0.01, *** 0.001 > *p*; *n* = 5). Dots are outliers.

In spring, the equilibration time for Ψ_stem_ determination differed among cultivars (Figure 2). For ‘Refošk’, wrapping leaves 30 or 10 min before collection caused a small underestimation of Ψ_stem_ (0.1 MPa, *p* < 0.001, Table S1), suggesting that at least 1 h equilibration time would be adequate for this variety. For ‘Merlot’ and ‘Pinot gris’, 10 min equilibration time was sufficient to obtain values comparable to those recorded in leaves equilibrated for 24 h, with the average Ψ_stem_ between −0.35 MPa and −0.20 MPa.

In summer, Ψ_stem_ was more negative than that measured at the beginning of the season, with average values of about −1.2 MPa, −1.8 MPa, −0.9 MPa and −1.5 MPa in ‘Refošk’, ‘Merlot’, ‘Pinot gris’ and ‘Merlot Kanthus^®^’, respectively (Figure 2). In this case, up to 1 h equilibration in ‘Refosk’ (*p* = 0.04) and 10 min in ‘Pinot gris’ (*p* = 0.054) and ‘Merlot’ (*p* = 0.01) would underestimate Ψ_stem_, but only by about 0.1 MPa. In ‘Merlot Kanthus^®^’, even 2 h bagging was not sufficient to equilibrate Ψ_stem_, which was about 0.2 MPa lower than that measured in leaves covered the day before collection (Figure 2d, *p* < 0.01).

### 2.3. LMA and LDMC

No difference was found in LMA (*p* = 0.13) and LDMC (*p* = 0.07) among the analysed cultivars (Table 1 and Table S2). However, both LMA and LDMC tended to be lower in ‘Pinot gris’ compared to the other cultivars.

## 3. Discussion

In this study, we tested two protocols for leaf and stem water potential determination on different grape cultivars in two timings of the growing season, in the view of maximizing the number of samples that can be sampled, stored and measured within limited daytime intervals. In particular, we determined the maximum tolerable storage time for leaf samples between harvest and Ψ_leaf_ measurement, and the minimum equilibration time between leaf bagging and harvest for Ψ_stem_ determination, in order to disentangle possible cultivar- and season-specific effects on the effectiveness of sampling protocols. Our data provide useful information to reconcile the need for accurate measurements with the desire to increase the number of samples measured per daytime.

The tests aimed at detecting the maximum possible storage time revealed that the cv. ‘Pinot gris’ was the most susceptible to relatively quick and marked drops in Ψ_leaf_ during storage in refrigerated bags. Indeed, a significant decrease in Ψ_leaf_ by 0.2 MPa was already observed 1 h after harvest, and by 0.4/0.5 MPa 3 h after harvest, both in the early and late season, corresponding to relatively well-watered and moderately stressed conditions, respectively (Figure 1). ‘Refošk’ was susceptible to significant Ψ_leaf_ drops after short storage times in the early season (1 h upon collection) or upon 2–3 h storage in the late season, but differences in Ψ_leaf_ with respect to time 0 were always very small (0.1 MPa). This difference could be considered tolerable when measuring water potentials with a Scholander bomb, and it is usually considered acceptable when equilibrating Ψ_leaf_ during experiments [17]. Conversely, ‘Merlot’ and ‘Merlot Kanthus^®^’ did not change their Ψ_leaf_ at any time interval and time of the season (Figure 1b,d), suggesting that storing leaves for some hours is acceptable when under proper storage conditions. Some studies suggest that some equilibration may be desirable before measuring the water potential of leaves that were actively transpiring when sampled (10 min [13]). Indeed, structural and related biophysical properties of leaf tissues can influence water movements inside the sample during equilibration of leaf water potential, due to hydraulic (dis)connection between the epidermis and veins [26,27]. In line with this concept, Rodriguez-Dominguez et al. [13] tested this effect in two species, *Olea Europaea* and *Eucalyptus camaldulensis*, finding out that the former needed at least 30 min equilibration time, compared to only 10 min time for the latter species. However, in transpiring *Olea europaea* leaves measured at midday, Ψ_leaf_ at leaf collection was higher (less negative), and the drop in Ψ_leaf_ was larger (i.e., was more negative) in leaves measured 30 min after collection. Therefore, it is possible that for some species or genotypes, e.g., in the variety ‘Pinot gris’, even under nontranspiring/moderately stressed conditions, the drop in water potentials can be progressive and relatively large even after short storage time. This indicates that even when following the most reliable sample preservation protocols [13], some water loss during storage in the bag might be unavoidable. This could be related to a lower leaf bulk capacitance, which can widely vary among species [28], or to differences in the bulk modulus of elasticity of leaf cells [29]. Moreover, this effect would be more likely to occur above the turgor loss point, as the capacitance is generally much lower before than after turgor loss [17]. Indeed, it should be noted that not only the variety, but also the plant water status (therefore, Ψ_leaf_ at time 0 in the present tests), can have an influence on maximum leaf storage time. In our study, ‘Merlot’ and ‘Merlot Kanthus^®^’ were the cvs. reaching the lowest Ψ_leaf_ values in the late-season campaign (ca. −1.7 MPa on average), and were those showing no significant storage effects. Ψ_leaf_ of ‘Refosk’ and ‘Pinot gris’ never fell below −1.2 MPa, so we do not know the storage effect at lower Ψ_leaf_ values. However, in the late season, Ψ_leaf_ at time 0 was similar in ‘Refosk’ and ‘Pinot gris’ (around −1.1 MPa on average), but Ψ_leaf_ of ‘Pinot gris’ dropped more strongly, and this drop was already significant at 1 h storage. Conversely, 2 h after collection, the drop in Ψ_leaf_ was just by 0.1 MPa in ‘Refosk’, while it was already by 0.4 MPa in ‘Pinot gris’. In this case, at comparable initial Ψ_leaf_, the cultivar was a key determinant in the maximum storage time selection.

Leaf area-specific capacitance is positively correlated to lamina thickness [30] and LMA [28]. Although we did not measure bulk leaf capacitance in the studied cultivars, we hypothesize that leaves of ‘Pinot gris’, with a tendency towards lower LMA than the other two cvs. (Table 2), had overall lower capacitance. This would mean that for the same water loss, a larger drop in Ψ_leaf_ would be expected in ‘Pinot gris’. This would explain the larger and significant drop in Ψ_leaf_ as a function of storage time observed in both early- and late-season tests for this cultivar. Future tests should focus on relating leaf structural properties with water potential drops during sample storage for water potential measurements.

The second test performed in this study aimed at determining the minimum equilibration time for Ψ_stem_ measurements, i.e., the mandatory time from leaf bagging to leaf sampling to correctly estimate Ψ_stem_. In a previous experiment, bagging time for the ‘Cabernet Sauvignon’ variety was 1 h [19], with no difference for leaves bagged for longer periods (2 and 6 h). Levin (2019) [11] performed water potential tests in the late growing season (post-veraison, before fruit harvest), when irrigation was stopped. The water potential values in that experiment were relatively low, suggesting a moderate water stress, and only 10 min equilibration was enough for accurate Ψ_stem_ determination. This result is in line with our measurements when performed post-veraison in all examined cultivars except ‘Merlot Kanthus^®^’, and with ‘Merlot’ and ‘Pinot gris’ in the early-season tests. Conversely, at least 1 h equilibration time was required for ‘Refošk’ in the early season (Figure 1a). Indeed, our tests performed in the early growing season suggest that when stomata are open due to the absence of physiological drought stress (i.e., drought stress induced by low soil water availability and/or high VPD), the required equilibration time for Ψ_stem_ measurements may be longer than 10 min, depending on the cultivar (as shown here in ‘Refošk’). On the other hand, stomatal closure in late summer could promote the substantial equilibration of leaf and stem water potential even before bagging, thus decreasing the minimum necessary equilibration time. Indeed, in ‘Refošk’, ‘Pinot gris’ and ‘Merlot’, the difference among Ψ_leaf_ and Ψ_stem_ measured at midday was higher in late spring (0.4 MPa on average) than in late summer (less than 0.1 MPa, on average, indicating almost complete stomatal closure). In ‘Merlot Kanthus^®^’, this Ψ_leaf_-Ψ_stem_ difference was by about 0.4 MPa, indicating partially open stomata. This could at least in part explain why even 2 h equilibration was not sufficient for correct Ψ_stem_ determination, causing a 0.2 MPa underestimation of Ψ_stem_.

In conclusion, our experiments indicate that careful tests for water potential determination must be performed on the plant material used for the experiments in order to improve measurement precision. These tests would be also pivotal in studies aimed at estimating plant water status from multispectral, environmental and thermal imaging data that use Ψ_stem_ as reference method for validation [31,32]. Given the different patterns found among cvs. and times of the season (which reflect the different water status of the plants among campaigns), this type of methodological care would be highly relevant if, for example, one wants to compare different species or cultivars and/or possible seasonal effects. Therefore, we propose the two protocols here presented as basic preliminary tests to be performed before real water potential measurements for experiments conducted both in the field and under controlled conditions, whether in crops or forest species.

## 4. Materials and Methods

### 4.1. Experimental Sites and Sampling Campaigns

The experiment was conducted by three different research groups in four different mature vineyards located in distinct geomorphological contexts and cultivated with different grapevine (*Vitis vinifera* L.) cultivars. In detail, we selected a ‘Refošk’ vineyard in Ceroglie in the Classical Karst (45.78509 N, 13.6407 E, Ceroglie Italy, University of Trieste team); a ‘Merlot’ vineyard located in a flysch terrace in Potok in the Vipava Valley (45.88194 N, 13.73458 E, 104 m a.s.l., Potok Slovenia, Agricultural Institute of Slovenia team); and a ‘Pinot gris’ and a ‘Merlot Kanthus^®^’ vineyard located in the alluvial Friulian plane (46°03′12″ N, 13°22′51″ E, 88 m a.s.l., Udine Italy, University of Udine team). ‘Refošk’ is extensively and typically cultivated in the cross-border area between Slovenia and Italy, as well as in Croatia (Istria) [33]. ‘Merlot Kanthus^®^’ is a patented cv. obtained by introgression of resistance genes to downy and powdery mildew (pedigree: Merlot × 20-3). ‘Merlot Kanthus^®^’ was registered in Italy in 2015, and because of this recent introduction, the surface planted with this variety is limited but increasing during the last years.

The tests (see below) were performed over two consecutive sunny days in two different times of the season, i.e., in late spring 2020 (25th–26th May), and at the end of summer (between end of August and first week of September). ‘Merlot Kanthus^®^’ was added later to the experiment, and therefore was only measured in summer. The summer measurements were conducted in 2020 for ‘Pinot gris’ and ‘Merlot Kanthus^®^’ (21st and 25th August), but were postponed in 2021 for ‘Refošk’ (6th and 7th September) and ‘Merlot’ (30th–31st August) due to low drought stress levels reached in summer 2020 in those vineyards. Indeed, ‘Merlot Kanthus^®^’ in 2020 was subjected to a rainfall exclusion experiment by covering the soil surface with plastic foils from the beginning of July to harvest time in September. ‘Pinot gris’ received both natural precipitation and irrigation in 2020. Vineyards cultivated with ‘Refošk’ and ‘Merlot’ received only natural precipitation.

Hourly air temperatures (T) and relative humidity (RH) for the study sites were taken from the available meteorological stations closest to the vineyards, i.e., Sgonico, Udine S.O. (www.osmer.fvg.it, accessed on 25 March 2022) and Bilje (https://meteo.arso.gov.si, accessed on 20 January 2022). Table 2 reports the average midday values for T and RH as recorded on the sampling dates in the central hours of the day (12–14 h, solar time, i.e., when leaves were harvested), together with the calculated VPD (kPa).

Water potential values were measured in the field on mature, fully expanded, sun-exposed leaves (collected between 12 and 14 h, solar time) with Scholander-type pressure chambers (model 1505D or 1000, PMS Instrument Co., Albany, OR, USA; model 3000F01, Soil Moisture Co., Santa Barbara, CA, USA). For each vineyard and sampling period, 5 plants were selected close to each other and sampled for both tests (see description below).

### 4.2. First Test: Time Span from Leaf Sampling to Water Potential Measurements

On each selected plant, four leaves were collected and immediately wrapped separately in cling film, taking care of also covering the whole petiole. One leaf was used for immediate Ψ_leaf_ measurement (control leaf at time 0). The other three leaves were stored in a zip-lock plastic bag containing a piece of humid paper to avoid water loss, and stored in a refrigerated bag, avoiding contact between the sample and ice blocks placed inside the bag. Sampling time for each plant was recorded. The stored leaves were measured 1, 2 and 3 h after sampling time (one per sampling time).

### 4.3. Second Test: Equilibration Time for Stem Minimum Water Potential Measurements

For each selected plant, one leaf was carefully wrapped in cling film and aluminium foil the evening before measurements (24 h, control), while the others 2 h, 1 h, 30 min and 10 min before sampling. At sampling time, all five leaves per plant were collected together, carefully sealed in plastic cling film and preserved in refrigerated bags, as described above, for Ψ_leaf_ procedure until measurements. Stem water potential (Ψ_stem_) was measured randomly, leaf by leaf, within the appropriate timing as determined by the first test experiment performed on the previous day.

### 4.4. LMA and LDMC

In September 2021, five additional leaves per cultivar were sampled, as described for Ψ_leaf_. Once in the laboratory, leaves were rehydrated in the dark for two hours to reach full turgor by immersing the cut petiole in distilled water and covering the leaves with cling film. Afterwards, leaf turgid weight was measured with an analytical balance after removing the petiole. Then, leaves were oven-dried for 24 h at 70 °C and the dry weight was measured. Leaf dry matter content (LDMC, mg g^−1^) was calculated as:

LDMC = leaf dry weight/leaf turgid weight,(1)

Leaf mass per area (LMA, g cm^−2^) was also calculated for the same leaves, by scanning the lamina with a scanner after measuring the fresh weight, as:

LMA = leaf dry weight/leaf area(2)

### 4.5. Statistical Analyses

Statistical analyses were carried out with R [34]. Boxplot panels were obtained with the “ggplot2” package. The effect of leaf storage time on Ψ_leaf_ (first test) and equilibration time for Ψ_stem_ measurements (second test) was tested by applying linear mixed-effects models (package “nlme”, “lme’” function), with the plant replicate as a random effect. Differences with respect to controls (0 h storage and 24 h leaf bagging for Ψ_leaf_ and Ψ_stem_ measurements, respectively) were tested by setting contrasts through the “trt.vs.ctrl” of the least-squares means “emmeans” function (package “emmeans”). LMA and LDMC differences among cvs., after verifying the validity of assumptions of normality of residuals and homogeneity of variances, were tested with one-way ANOVA tests through the “aov” function, followed by Tukey’s honestly significant differences post hoc test if ANOVA was significant (α = 0.05) through the “TukeyHSD” function in the “stats” package.

## Figures and Tables

**Table 1:** Leaf mass per area (LMA) and leaf dry matter content (LDMC) measured on the grapevine cultivars. Values are means ± standard error. Different letters indicate significant differences among cultivars (*p* < 0.05, *n* = 5).

	‘Refošk’	‘Pinot Gris’	‘Merlot Kanthus^®^’
LMA (g m^−2^)	81.7 ± 3.0 ^a^	70.6 ± 5.4 ^a^	77.2 ± 2.2 ^a^
LDMC (mg g^−1^)	355.5 ± 7.7 ^a^	318.3 ± 18.6 ^a^	360.0 ± 9.2 ^a^

**Table 2:** Average ± SD midday temperature (T), relative humidity (RH) and vapour pressure deficit (VPD) measured in the central hours of the day in the test dates in the meteorological stations closest to the study vineyards. Note that ‘Pinot gris’ and ‘Merlot Kanthus^®^’ vineyards were in the same location.

	Early-Season Tests			Late-Season Tests		
Cultivar	T (°C)	RH (%)	VPD (kPa)	T (°C)	RH (%)	VPD (kPa)
‘Refošk’	19.7 ± 0.4	46 ± 3	1.26 ± 0.09	26.4 ± 0.3	33 ± 1	2.31 ± 0.06
‘Merlot’	21.3 ± 0.2	41 ± 2	1.53 ± 0.06	22.6 ± 0.3	55 ± 1	1.23 ± 0.02
‘Pinot gris’ ‘Merlot Kanthus^®^’	22.1 ± 0.3	47 ± 1	1.40 ± 0.05	28.7 ± 0.5	47 ± 4	2.08 ± 0.13

## Data Availability

The data presented in this study are available on request from the corresponding author.

## Supplementary Materials

The following supporting information can be downloaded at: https://www.mdpi.com/article/10.3390/plants12132412/s1, Table S1: Summary of the one-way Anova/GLS model for the analysed parameters in the tests (1st and 2nd, see article text) in the different vineyards and campaigns (early season, late season); Table S2: Summary of the one-way Anova/GLS model for leaf mass per area (LMA) and leaf dry matter content (LDMC) detecting differences among cultivars ‘Refošk’, ‘Pinot gris’ and ‘Merlot Kanthus^®^’.

## References

[1] Boyer J.S., Knipling E.B. (1965). Isopiestic technique for measuring leaf water potentials in leaves with a thermocouple psychrometer. Proc. Nat. Acad. Sci. USA.

[2] Brunetti C., Alderotti F., Pasquini D., Stella C., Gori A., Ferrini F., Righele M., Centritto M. (2022). On-line monitoring of plant water status: Validation of a novel sensor based on photon attenuation of radiation through the leaf. Sci. Tot. Env..

[3] Jain P., Liu W., Zhu S., Chang C.Y.-Y., Melkonian J., Rockwell F.E., Pauli D., Sun Y., Zipfel W.R., Holbrook N.M. (2021). A minimally disruptive method for measuring water potential in planta using hydrogel nanoreporters. Proc. Nat. Acad. Sci. USA.

[4] Turner N.C. (1981). Techniques and experimental approaches for the measurement of plant water status. Plant Soil.

[5] Scholander P.F., Bradstreet E.D., Hemmingsen E.A., Hammel H.T. (1965). Sap pressure in vascular plants: Negative hydrostatic pressure can be measured in plants. Science.

[6] Fernández J.E. (2017). Plant-based methods for irrigation scheduling of woody crops. Horticulturae.

[7] Gambetta G.A., Herrera J.C., Dayer S., Feng Q., Hochberg U., Castellarin S.D. (2020). Grapevine drought stress physiology: Towards an integrative definition of drought tolerance. J. Exp. Bot..

[8] Petruzzellis F., Natale S., Bariviera L., Calderan A., Mihelčič A., Reščič J., Sivilotti P., Šuklje K., Lisjak K., Vanzo A. (2022). High spatial heterogeneity of water stress levels in Refošk grapevines cultivated in Classical Karst. Agr. Water Manag..

[9] Nardini A., Casolo V., Dal Borgo A., Savi T., Stenni B., Bertoncin P., Zini L., McDowell N.G. (2016). Rooting depth, water relations and non-structural carbohydrate dynamics in three woody angiosperms differentially affected by an extreme summer drought. Plant Cell Environ..

[10] Nardini A., Petruzzellis F., Marusig D., Tomasella M., Natale S., Altobelli A., Calligaris C., Floriddia G., Cucchi F., Forte E. (2021). Water ‘on the rocks’: A summer drink for thirsty trees?. New Phytol..

[11] Levin A.D. (2019). Re-evaluating pressure chamber methods of water status determination in field-grown grapevine (*Vitis* spp.). Agr. Water Manag..

[12] Hochberg U. (2020). Facilitating protocols while maintaining accuracy in grapevine pressure chamber measurements-comments on Levin 2019. Agr. Water Manag..

[13] Rodriguez-Dominguez C.M., Forner A., Martorell S., Choat B., Lopez R., Peters J.M.R., Pfautsch S., Mayr S., Carins-Murphy M.R., McAdam S.A.M. (2022). Leaf water potential measurements using the pressure chamber: Synthetic testing of assumptions towards best practices for precision and accuracy. Plant Cell Environ..

[14] Wu J., Serbin S.P., Ely K.S., Wolfe B.T., Dickman L.T., Grossiord C., Michaletz S.T., Collins A.D., Detto M., McDowell N.G. (2020). The response of stomatal conductance to seasonal drought in tropical forests. Glob. Chang. Biol..

[15] Sack L., Holbrook N.M. (2006). Leaf hydraulics. Annu. Rev. Plant Biol..

[16] Sorek Y., Greenstein S., Netzer Y., Shtein I., Jansen S., Hochberg U. (2021). An increase in xylem embolism resistance of grapevine leaves during the growing season is coordinated with stomatal regulation, turgor loss point and intervessel pit membranes. New Phytol..

[17] Brodribb T.J., Holbrook N.M. (2003). Stomatal closure during leaf dehydration, correlation with other leaf physiological traits. Plant Physiol..

[18] Santesteban L., Miranda C., Marín D., Sesma B., Intrigliolo D., Mirás-Avalos J., Escalona J., Montoro A., de Herralde F., Baeza P. (2019). Discrimination ability of leaf and stem water potential at different times of the day through a meta-analysis in grapevine (*Vitis vinifera* L.). Agr. Water Manag..

[19] Choné X., Van Leeuwen C., Dubourdieu D., Gaudillère J.P. (2001). Stem water potential is a sensitive indicator of grapevine water status. Ann. Bot..

[20] Naor A. (2000). Midday stem water potential as a plant water stress indicator for irrigation scheduling in fruit trees. Acta Hortic..

[21] Calderan A., Sivilotti P., Braidotti R., Mihelčič A., Lisjak K., Vanzo A. (2021). Managing moderate water deficit increased anthocyanin concentration and proanthocyanidin galloylation in “Refošk” grapes in Northeast Italy. Agr. Water Manag..

[22] Herrera J., Bucchetti B., Sabbatini P., Comuzzo P., Zulini L., Vecchione A., Peterlunger E., Castellarin S. (2015). Water deficit and severe trimming effect on Merlot. Aust. J. Grape Wine R..

[23] Rockwell F.E., Holbrook N.M., Zwieniecki M.A. (2011). Hydraulic conductivity of red oak (*Quercus rubra* L.) leaf tissue does not respond to light. Plant Cell Environ..

[24] Fulton A., Buchner R., Gilles C., Olson B., Bertagna N., Walton J., Schwankl L., Shackel K. (2002). Rapid equilibration of leaf and stem water potential under field conditions in almonds, walnuts, and prunes. Horttechnology.

[25] Lavoie-Lamoureux A., Sacco D., Risse P.-A., Lovisolo C. (2017). Factors influencing stomatal conductance in response to water availability in grapevine: A meta-analysis. Physiol. Plantarum.

[26] Buckley T.N., John G.P., Scoffoni C., Sack L. (2015). How does leaf anatomy influence water transport outside the xylem?. Plant Physiol..

[27] Zwieniecki M.A., Brodribb T.J., Holbrook N.M. (2007). Hydraulic design of leaves: Insights from rehydration kinetics. Plant Cell Environ..

[28] Blackman C.J., Brodribb T.J. (2011). Two measures of leaf capacitance: Insights into the water transport pathway and hydraulic conductance in leaves. Funct. Plant Biol..

[29] Patakas A., Noitsakis B. (1997). Cell wall elasticity as a mechanism to maintain favorable water relations during leaf ontogeny in grapevines. Am. J. Enol. Viticult..

[30] Sack L., Cowan P.D., Jaikumar N., Holbrook N.M. (2003). The ‘hydrology’ of leaves: Co-ordination of structure and function in temperate woody species. Plant Cell Environ..

[31] Gutiérrez S., Fernández-Novales J., Diago M.P., Iñiguez R., Tardaguila J. (2021). Assessing and mapping vineyard water status using a ground mobile thermal imaging platform. Irrig. Sci..

[32] Diago M.P., Tardaguila J., Barrio I., Fernández-Novales J. (2022). Combination of multispectral imagery, environmental data and thermography for on-the-go monitoring of the grapevine water status in commercial vineyards. Eur. J. Agron..

[33] Kozjak P., Korošec-Koruza Z., Javornik B. (2003). Characterisation of cv. Refošk (*Vitis vinifera* L.) by SSR markers. Vitis.

[34] (2019). R Core Team R: A Language and Environment for Statistical Computing. R Foundation for Statistical Computing, Vienna, Austria. https://www.R-project.org/.

