# Supplementary material for: Best Procedures for Leaf and Stem Water Potential Measurements in Grapevine: Cultivar and Water Status Matter
Source: Plants (Basel). 2023 Jun 22;12(13):2412. doi: 10.3390/plants12132412 (PMC10346302; doi:10.3390/plants12132412)
Supplement: Supplementary file 1 [file plants-12-02412-s001.zip › plants-2462698-supplementary.pdf]

Table S1 - Summary of the Estimated Marginal Means (Least Square Means) for the Linear Mixed-Effects Models applied, setting comparison among the control (0h or 24h for the 1st and 2nd experiment, respectively) and the other timings in the tests (1st and 2nd, see article text) in the different cultivars and campaigns (early season, late season).

Comparison	estimate	SE	df	T ratio	*P* value	Significance
**1st test** -'Refošk' – early season						
1h - 0h	-0.09	0.0269	12	-3.343	0.0158	*
2h - 0h	-0.15	0.0269	12	-5.571	0.0003	***
3h - 0h	-0.23	0.0269	12	-8.542	<0.0001	***
**1st test** -'Merlot' – early season						
1h - 0h	-0.07	0.0596	12	-1.175	0.5261	
2h - 0h	-0.08	0.0596	12	-1.343	0.4320	
3h - 0h	-0.16	0.0596	12	-2.685	0.0518	
**1st test** –'Pinot gris' – early season						
1h - 0h	-0.216	0.0237	12	-9.128	0.5261	***
2h - 0h	-0.324	0.0237	12	-13.691	0.4320	***
3h - 0h	-0.396	0.0237	12	-16.734	0.0518	***
**1st test** –'Refošk' – late season						
1h - 0h	-0.06	0.0362	12	-1.659	0.2820	
2h - 0h	-0.11	0.0362	12	-3.041	0.0273	*
3h - 0h	-0.14	0.0362	12	-3.871	0.0061	**
**1st test** -'Merlot' – late season						
1h - 0h	0.05	0.0634	12	0.788	0.7547	
2h - 0h	0.09	0.0634	12	1.419	0.3923	
3h - 0h	0.10	0.0634	12	1.576	0.3172	
**1st test** –'Pinot gris' – late season						
1h - 0h	-0.220	0.0458	12	-4.799	0.0012	**
2h - 0h	-0.396	0.0458	12	-8.639	<.0001	***
3h - 0h	-0.504	0.0458	12	-10.995	<.0001	***
**1st test** –'Merlot Kanthus®' – late season						
1h - 0h	-0.028	0.0873	12	-0.321	0.9585	
2h - 0h	0	0.0873	12	0.000	1.0000	
3h - 0h	-0.068	0.0873	12	-0.779	0.7601	
**2nd test** -'Refošk' – early season						
2h - 24h	-0.046	0.0225	16	-2.044	0.1756	
1h - 24h	-0.030	0.0225	16	-1.333	0.4946	
30min - 24h	-0.110	0.0225	16	-4.888	0.0006	***
10min – 24h	-0.110	0.0225	16	-4.888	0.0006	***
**2nd test** -'Merlot' – early season						
2h - 24h	0.05	0.0286	16	1.746	0.2832	
1h - 24h	0.02	0.0286	16	0.698	0.8545	
30min - 24h	-0.02	0.0286	16	-0.698	0.8545	
10min – 24h	0.00	0.0286	16	0.000	1.0000	
**2nd test** –'Pinot gris' – early season						
2h - 24h	-0.012	0.0122	16	-0.981	0.7047	
1h - 24h	-0.016	0.0122	16	-1.308	0.5092	
30min - 24h	-0.024	0.0122	16	-1.962	0.2013	
10min – 24h	-0.020	0.0122	16	-1.635	0.3333	
**2nd test** –'Refošk' – late season						

2h - 24h	-0.03	0.0283	16	-1.061	0.6576	
1h - 24h	-0.08	0.0283	16	-2.828	0.0410	*
30min - 24h	-0.10	0.0283	16	-3.536	0.0098	**
10min – 24h	-0.09	0.0283	16	-3.182	0.0202	*
**2nd test** -‘Merlot’ – late season						
2h - 24h	0.02	0.0414	16	0.483	0.9368	
1h - 24h	0.01	0.0414	16	0.241	0.9876	
30min - 24h	-0.05	0.0414	16	-1.207	0.5692	
10min – 24h	-0.14	0.0414	16	-3.381	0.0134	*
**2nd test** –‘Pinot gris’ – late season						
2h - 24h	-0.064	0.0373	16	-1.718	0.2955	
1h - 24h	-0.056	0.0373	16	-1.503	0.3998	
30min - 24h	-0.080	0.0373	16	-2.147	0.1470	
10min – 24h	-0.100	0.0373	16	-2.684	0.0544	
**2nd test** –‘Merlot Kanthus®’ – late season						
2h - 24h	-0.196	0.0455	16	-4.306	0.0020	**
1h - 24h	-0.188	0.0455	16	-4.130	0.0029	**
30min - 24h	-0.164	0.0455	16	-3.603	0.0085	**
10min – 24h	-0.172	0.0455	16	-3.779	0.0059	**

Significance codes: 0 '***' 0.001 '**' 0.01 '*' 0.05

Table S2 - Summary of the one-way Anova/GLS model for leaf mass per area (LMA) and leaf dry matter content (LDMC) detecting differences among cultivars ‘Refošk’, ‘Pinot gris’ and ‘Merlot Kanthus®’.

	Df	Sum Sq	Mean Sq	F value	Pr(>F)	Significance
**LMA**	2	310.8	155.38	2.145	0.16	
Residuals	12	869.3	72.44			
**LDMC**	2	5247	2623.3	3.212	0.0763	.
Residuals	12	9800	816.7			
